# *In vivo* spectroscopy and NMR metabolite fingerprinting approaches to connect the dynamics of photosynthetic and metabolic phenotypes in resurrection plant *Haberlea rhodopensis* during desiccation and recovery

**DOI:** 10.3389/fpls.2015.00564

**Published:** 2015-07-21

**Authors:** Petko Mladenov, Giovanni Finazzi, Richard Bligny, Daniela Moyankova, Diana Zasheva, Anne-Marie Boisson, Sabine Brugière, Vasilena Krasteva, Kalina Alipieva, Svetlana Simova, Magdalena Tchorbadjieva, Vasiliy Goltsev, Myriam Ferro, Norbert Rolland, Dimitar Djilianov

**Affiliations:** ^1^Abiotic Stress Group, Agrobioinstitute, Agricultural AcademySofia, Bulgaria; ^2^Laboratoire de Physiologie Cellulaire et Végétale, Institut de Recherches en Technologies et Sciences pour le Vivant, CEA, CNRS, Université Grenoble AlpesINRA, Grenoble, France; ^3^Institute of Biology and Immunology of Reproduction, Bulgarian Academy of SciencesSofia, Bulgaria; ^4^Laboratoire de Biologie à Grande Echelle, Institut de Recherches en Technologies et Sciences pour le Vivant, CEA, Université Grenoble AlpesINSERM, Grenoble, France; ^5^Department of Biophysics and Radiobiology, Faculty of Biology, Sofia UniversitySofia, Bulgaria; ^6^Laboratory “Nuclear Magnetic Resonance", Institute of Organic Chemistry with Centre of Phytochemistry, Bulgarian Academy of SciencesSofia, Bulgaria; ^7^Department of Biochemistry, Faculty of Biology, Sofia UniversitySofia, Bulgaria

**Keywords:** resurrection plant, drought stress, photosynthesis, metabolism, phenotype, *Haberlea rhodopensis*

## Abstract

The resurrection plant *Haberlea rhodopensis* was used to study dynamics of drought response of photosynthetic machinery parallel with changes in primary metabolism. A relation between leaf water content and photosynthetic performance was established, enabling us to perform a non-destructive evaluation of the plant water status during stress. Spectroscopic analysis of photosynthesis indicated that, at variance with linear electron flow (LEF) involving photosystem (PS) I and II, cyclic electron flow around PSI remains active till almost full dry state at the expense of the LEF, due to the changed protein organization of photosynthetic apparatus. We suggest that, this activity could have a photoprotective role and prevent a complete drop in adenosine triphosphate (ATP), in the absence of LEF, to fuel specific energy-dependent processes necessary for the survival of the plant, during the late states of desiccation. The NMR fingerprint shows the significant metabolic changes in several pathways. Due to the declining of LEF accompanied by biosynthetic reactions during desiccation, a reduction of the ATP pool during drought was observed, which was fully and quickly recovered after plants rehydration. We found a decline of valine accompanied by lipid degradation during stress, likely to provide alternative carbon sources for sucrose accumulation at late stages of desiccation. This accumulation, as well as the increased levels of glycerophosphodiesters during drought stress could provide osmoprotection to the cells.

## Introduction

Growth and yield of crop plants is severely hampered by drought. Water deficit triggers loss of cellular turgor, disruption of water potential gradients, production of reactive oxygen species (ROS), changes in cell volume and membrane integrity, denaturation of proteins, etc. (Lawlor and Cornic, 2002; Wullschleger et al., 2002; Osakabe et al., 2014). Plants have evolved various mechanisms to cope with drought stress, which are currently studied at the level of gene expression, abundance, and posttranslational modifications of proteins and changes in metabolite contents (Dinakar and Bartels, 2013; Deshmukh et al., 2014). On the other hand, recently developed time-resolved spectroscopic techniques to assess photosynthesis function *in vivo* could provide a bridge between the core dynamics of photosynthetic processes and the changes in the plant transcriptome, proteome, or metabolome (Baker et al., 2007; Eberhard et al., 2008; Pinheiro and Chaves, 2011). Recently, characterization of fast fluorescence emission yield has been employed to evaluate the response of plants to drought in a non-destructive manner (Strasser et al., 2010; Goltsev et al., 2012).

Resurrection plants are a small group of plants, represented in various taxonomic groups, from pteridophytes to dicotyledons, distributed in almost all continents (Scott, 2000), with ability to survive almost complete dehydration of their vegetative tissues. After re-watering, they rapidly restore a fully active state, which makes them unique models to study plant tolerance to severe water deficit. Similar to desiccation sensitive plant species, in resurrection plants the decrease of water potential and stomatal conductance during drought stress begin with the closure of stomata and inhibition of photo-carbon reaction (Georgieva et al., 2007; Tuba and Lichtenthaler, 2011; Dinakar et al., 2012). Later, as a response to water depletion, the desiccation tolerant species replace the water with accumulation of osmolytes and compounds with ROS scavenging functions (Moore et al., 2005; Peters et al., 2007; Bartels and Hussain, 2011; Jovanović et al., 2011; Oliver et al., 2011; Yobi et al., 2013; Suguiyama et al., 2014). In addition, several classes of drought-protective proteins have been reported in resurrection plants (Dinakar and Bartels, 2013). Thus, a general picture of water stress responses in resurrection plants has been proposed, where downregulation of photo-carbon reaction, coupled to respiration allows lowering light stress, while maintaining carbon supply for *de novo* accumulation of protective compounds to ensure survival of the anhydrobiotes in dry state (Leprince and Hoekstra, 1998; Avelange-Macherel et al., 2006). Indeed, the increasing of the levels of some enzymes involved in glycolysis during drought indicates a shift between autotrophy and heterotrophy during desiccation in resurrection plants (Griffiths et al., 2014).

*Haberlea rhodopensis* (Gesneriacea) is a Balkan endemite and glacial relict and, together with *Ramonda* sp., are the only resurrection plant species in Europe. Both genera are homoiochlorophyllous desiccation-tolerant plants – they undergo reversible modifications in the supermolecular structure of the photosynthetic machinery during desiccation, instead of degrading the chlorophyll pool. Indeed, our previous work showed high molecular organization of photosynthetic apparatus (Mladenov et al., 2013), which is flexible in both shade and sun populations of plants during desiccation (Sárvária et al., 2014). Besides decreasing the Photosystem II (PSII) functionality, CO_2_ fixation and starch content during the early stages of desiccation (Muller et al., 1997; Georgieva et al., 2007), pronounced accumulation of sucrose and polyphenols has been observed during the last stages of desiccation (Berkov et al., 2011; Djilianov et al., 2011; Gechev et al., 2013; Moyankova et al., 2014). This suggests activation of anabolic reactions during the last stages of drought, in parallel with the inhibition of the photo carbon reaction. Recently, heat shock proteins have been proposed to play protective and stabilizing role on the cellular structures in *H. rhodopensis* during severe water deficit (Gechev et al., 2013).

Here, we used the resurrection plant *H. rhodopensis* to evaluate the dynamics of drought-induced response of photosynthetic machinery and changes in primary metabolism during desiccation and recovery. *In vivo* fast fluorescence-based approach was applied to study the relation between leaf water content (WC) and photosynthetic performance at stress and recovery. This enabled us to establish and further perform a non-destructive evaluation of stress response, based on the changes in the photosynthetic reaction. We then applied absorption spectroscopy and BN-PAGE to quantify the efficiency of linear electron flow (LEF) and cyclic electron flow (CEF) and the protein organization of photosynthetic apparatus. The ^13^C and ^31^P NMR spectroscopy allowed us to measure the changes in energy metabolites, glycolytic intermediates, and metabolites involved in lipid and sugar metabolism, in parallel with the changes of photosynthetic performance. Next we applied ^1^H NMR metabolic fingerprinting to evaluate changes in amino acid, sugar, and respiratory metabolism at selected states of drought and recovery.

## Materials and Methods

### Plant Material and Stress Treatments

*Haberlea rhodopensis* plants were routinely regenerated and propagated *in vitro* (Djilianov et al., 2005) and then transferred to soil. The plants were grown in well-watered pots in a controlled environment at 24°C day and night, a 16-h photoperiod, 40–60% relative humidity, and a photon flux density of 36 μmol m^-2^ s^-1^ for about a year. Dehydration was imposed by withholding water. After achieving a fully desiccated state, rehydration was started with automated plant watering box (Gardena GMbH Germany) at programmed regime of irrigation. The leaf samples for the parallel measurements of WC and prompt chlorophyll fluorescence (three leaves per plant, from three different plants for each PEA device) were harvested at the beginning of dehydration from irrigated plants, and then every day during the course of dehydration. The leaf samples for recovery were collected at 6, 12, 36, 48, 72, and 168 h after rewatering of the plants. Leaf WC was calculated using the following formula: WC (%) = (FW–DW) × 100/FW, where FW is fresh weight and DW is dry weight of leaves dried at 80°C for 48 h.

### JIP Test

Two independent experiments were performed using both Handy- and Multi-Plant efficiency analyzers (PEA; Hansatech Instrument Ltd., King’s Lynn, Norfolk, PE30 4NE, UK) devices to apply the JIP test analysis on leaves of dark-adapted *H. rhodopensis* plants. In addition, only Handy PEA device was used for the prompt chlorophyll *a* fluorescence on plants in light-adapted state. The JIP-test equations that connect the shape of photoinduced fluorescence rise with important structural and functional photosynthetic parameters are based on the Theory of Energy Fluxes in Biomembranes (Strasser, 1978; Strasser et al., 2004). The definitions and equations used here are derived from the well-known Z scheme of photosynthesis, expressed by sequential energy fluxes. Intermediate energy fluxes are the trapping flux, defined as the energy flux leading to the reduction of pheophytine and Q_A_, and the electron transport flux that refers to the electron transport further than Q_A_. At each step, the energy influx is bifurcated to an outflux for energy conservation *via* electron transfer and an outflux for dissipation. Measurements were conducted for 1 s induction period. Leaves were illuminated by red light of 2000 μmol m^-2^ s^-1^ PPFD after 2 h dark adaptation. For measurements in the light-adapted state, 10 min additional illumination by 50 μmol m^-2^ s^-1^ actinic light was applied, after the measurement of fluorescence on leaves in dark-adapted state.

After quantification of water stress and evaluation of intermediate states with both devices, we used only handy PEA for the further estimation of the plant stress status in all other experiments. We also used the protocol for light-adapted plants, mentioned above, to compare the JIP test parameters with the rates of LEF, measured in the same leaf.

### Spectroscopic Measurements

In parallel to the JIP assays with PEA, we also performed *in vivo* spectroscopy using a Joliot Type Spectrometer (JTS10; Biologic Grenoble, France) on leaves in different stages of desiccation. To determine the efficiency of electron flow, we measured changes in the redox changes of the PSI primary electron donor (P_700_) upon illumination with weak far red light as described previously (Joliot and Joliot, 2005). Actinic far-red illumination was provided by a LED peaking at 720 nm, filtered through three Wratten filters 55 that block wavelengths shorter than 700 nm. When needed, the maximum extent of P_700_^+^ was estimated from the kinetics of P_700_ oxidation as in Joliot and Joliot (2005). The relative efficiency of linear and CEF in different conditions was obtained by comparing P_700_redox changes with fluorescence derived parameters reflecting PSII activity as explained in the text. These parameters were evaluated using the same instrument in the “fluorescence” mode. The maximum fluorescence emission in the dark-adapted state (2 h in dark), *Fm*, and in the light-adapted state (10 min at 37 μE), *Fm*′ were measured by applying a pulse of white light. The effective photochemical quantum yield of PSII was measured as PSII yield [Y (II) = (*Fm*′-*F*)/*Fm*′], where *Fm*′ is the maximum fluorescence emission and *F* is the steady-state level of fluorescence.

### Preparation of Thylakoids and BN-PAGE Analysis

Five grams of leaves were harvested at each stage of stress according to the measured CEF to LEF ratio. Then, the leaves were homogenized with Warring blender three times for 2 s in 30 ml ice-cold grinding buffer (0.33 M sucrose, 10 mM Tricine, 30 mM MOPS-KOH pH 7.9, 5 mM ethylenediamine-tetraacetic acid (EDTA), 10 mM NaHCO_3_, 2 mM MgCl_2_, 0.1% (w/v) BSA, 2 mM DTT, 20 mM ascorbate, 1% (w/v) polyvinylpyrrolidone (PVP 40). The homogenate was filtered through nylon mesh (20 μm pore size) and three layers of Miracloth, and then centrifuged at 1500*g* for 6 min at 4°C. The pellet was gently resuspended in washing buffer (Seigneurin-Berny et al., 2008) containing: 0.33 M sucrose, 1.25 mM EDTA, 2.5 mM MgCl_2_, 10 mM Tricine, 30 mM MOPS-KOH pH 7.8 and then centrifuged at 1500*g* for 6 min at 4°C. The entire washing step was performed twice. After the second centrifugation, the pellet was resuspended in 2 ml washing buffer and loaded onto 90 and 20% two-step Percoll gradients in washing buffer. The resulted green band between the layers was collected and washed twice in washing buffer with centrifugation at 1500*g* for 6 min at 4°C. The pellet was resuspended in hypotonic buffer for disruption of intact chloroplasts and then stored in liquid nitrogen. An aliquot of thylakoids corresponding to 750 μg protein was centrifuged at 8000*g* for 6 min at 4°C. The pellet was resuspended in 83 μl BN-PAGE solubilization buffer (Wittig et al., 2006) with 10 μl 50% (v/v) glycerol. Then, 7 μl of 20% (w/v) DDM (*n*-dodecyl-β-D-maltoside) was added to give final concentration of 1.5% in solubilization buffer and 1.65 g/g detergent/protein ratio and centrifuged at 18,000*g* for 15 min at 4°C. The insolubilized pellet was discarded and the supernatant containing solubilized grana thylakoids was transferred to a new tube. The protein complexes were then separated using BN-PAGE according to Wittig et al. (2006).

### ^13^C and ^31^P NMR Analyses of Perchloric Acid Extracts

Five grams of leaves (fresh weight) for each stress stage in the light-adapted state were quickly frozen in liquid nitrogen as previously described (Aubert et al., 2007). Next, they were ground to a fine powder with 0.5 ml of 70% (v/v) perchloric acid and internal standards. After addition of 5 ml of 7% (v/v) perchloric acid the suspension was centrifuged at 15,000*g* for 10 min to remove particulate matter and the supernatant was buffered with 2 M KHCO_3_ to pH 5.2. The supernatant was then centrifuged at 10,000*g* for 10 min to remove KClO_4_ and the supernatant was lyophilized. The freeze-dried material was dissolved in 2.5 ml H_2_O containing 10% (v/v) D_2_O, and stored frozen. Spectra were recorded on a Bruker NMR spectrometer equipped with 10 mm multinuclear probe tuned at 162.0 or 100.6 MHz for ^31^P or ^13^C NMR studies, respectively. The deuterium resonance of D_2_O was used as a lock signal. ^13^C-NMR acquisition conditions: 90° radio frequency pulses (19 μs) at 6 s intervals; spectral width 20,000 Hz; 3,600 scans; Waltz-16 ^1^H decoupling sequence (with two levels of decoupling: 2.5 W during acquisition time, 0.5 W during delay). Free induction decays were collected as 16-K data points, zero filed to 32 K and processed with a 0.2 Hz exponential line broadening. ^13^C-NMR spectra were referenced to hexamethyldisiloxane at 2.7 ppm. The pH was adjusted to 7.5. ^31^P-NMR acquisition conditions: 70° radio frequency pulses (15 μs) at 3.6 s intervals; spectral width 8200 Hz; 4096 scans Waltz-16 ^1^H decoupling sequence (with two levels of decoupling: 1 W during acquisition time, 0.5 W during delay). Free induction decays were collected as 8-K data points; zero filed to 16 K and processed with a 0.2 Hz exponential line broadening. The pH was adjusted to 7.5. The assignments and quantification of total adenosine diphosphate (ADP) and adenosine triphosphate (ATP) was done in Principal Component Analysis (PCA) extracts as previously described (Roby et al., 1987; Aubert et al., 2007; Gout et al., 2014). Identified compounds were quantified by comparison of the integral of resonance peaks of standards added to the samples before grinding. The utilized standards were methylphosphonate and maleate for ^31^P and ^13^C NMR analyses, respectively. For each state of drought stress triplicates were used for perchloric acid extraction and spectra recordings.

### ^1^H NMR Analyses of Methanol/Water Extracts

Lyophilized leaves (130 mg) for each stress stage were homogenized with 1 ml of tetradeuteromethanol/deuterium oxide in ratio 1/1. The obtained homogenate was sonicated for 20 min followed by centrifugation at 15,000*g* for 20 min. The resulted supernatant was then transferred to a new tube. Six hundred microliters were used for the ^1^H NMR analysis. Spectra were recorded on an AVANCE II+ Bruker NMR spectrometer using a 5 mm BBO probe tuned at 600.1 and 150.9 MHz for ^1^H or ^13^C nuclei, respectively. The deuterium resonance of deuteromethanol was used as a lock signal. ^1^H-NMR acquisition conditions: 30° radio frequency pulses (3.6 μs) at 4 s interval; spectral width 6,361 Hz; 256 scans; ^1^H presaturation of the D_2_O signal for 1.5 s has been performed. Free induction decays were collected as 32-K data points, zero filled to 64 K and processed with a 0.3 Hz exponential line broadening. ^1^H-NMR spectra were referenced and quantified to (Trimethylsilyl) propionic-d_4_ acid Na salt at 0 ppm. J-resolved, DQF-COSY and HSQC 2D spectra have been acquired for assignment and/or verification of the proton signals (Mustafa et al., 2011). For each state of drought stress, triplicates were used for extraction and spectra recordings.

### Statistical Analyses

The recordings from both handy PEA and M-PEA were extracted with handy PEA and M-PEA Data Analyzers. Then, in order to cluster the leaf samples according to the JIP TEST parameters, we used PCA. The analyzed covariance matrix was formed by the basic JIP-TEST variables of the individual values of the leaf disks sampled during desiccation and recovery, respectively, for both devices. The data were standardized to zero mean and unit variance. Then regression analysis for correlation between JIP-TEST parameters and leaf WC was performed. To classify the stress states according to the similarity of JIP parameters, we clustered the scores from the first tree components of PCA under a typical self organizing map (SOM) scheme (neural network toolbox MatLab). A 2-by-2 hexagonal one-dimensional SOM of four neurons was used. The input vectors were mapped in k-dimensional space in which the *i*th coordinate represent the PC1, 2 and 3 scores of the *i*th sample. Thus, 150 leaf samples for desiccation and 75 for recovery were represented by three elements each (PC1, PC2, PC3) as the input vectors. Two hundred training iterations were used during clustering, with distances calculated according to the Manhattan distance neighborhood function (neural network toolbox, MatLab). First, ANOVA was used to determine the statistical reliability of the differences of each metabolite during the treatments. Then, PCA was performed in order to summarize the variance of metabolic data during the treatments. The analyzed data matrix contains all treatments and the respective replicates (*n* = 3) as rows and metabolites as columns. Prior to PCA, the data matrices were log2 transformed and standardized to zero mean and unit variance. In addition, for better visualization and clustering of the metabolite dynamics, Hierarchical cluster analysis (HCA) /HeatMap analysis was performed. All data were analyzed with MatLab software according to standard procedures. For the visualization of metabolic changes in the context of the plant metabolism, we first mapped all metabolites in AraCyc database, and then used these metabolic pathways as templates, subsequently merged and edited with Cytoscape software. The relative values of each treatment (to the values of fully hydrated leafs) then were drawn and analyzed for correlation by Omics Analyzer plug-in for Cytoscape.

## Results

### Evaluation of Leaf Water Status

**FIGURE 1:**
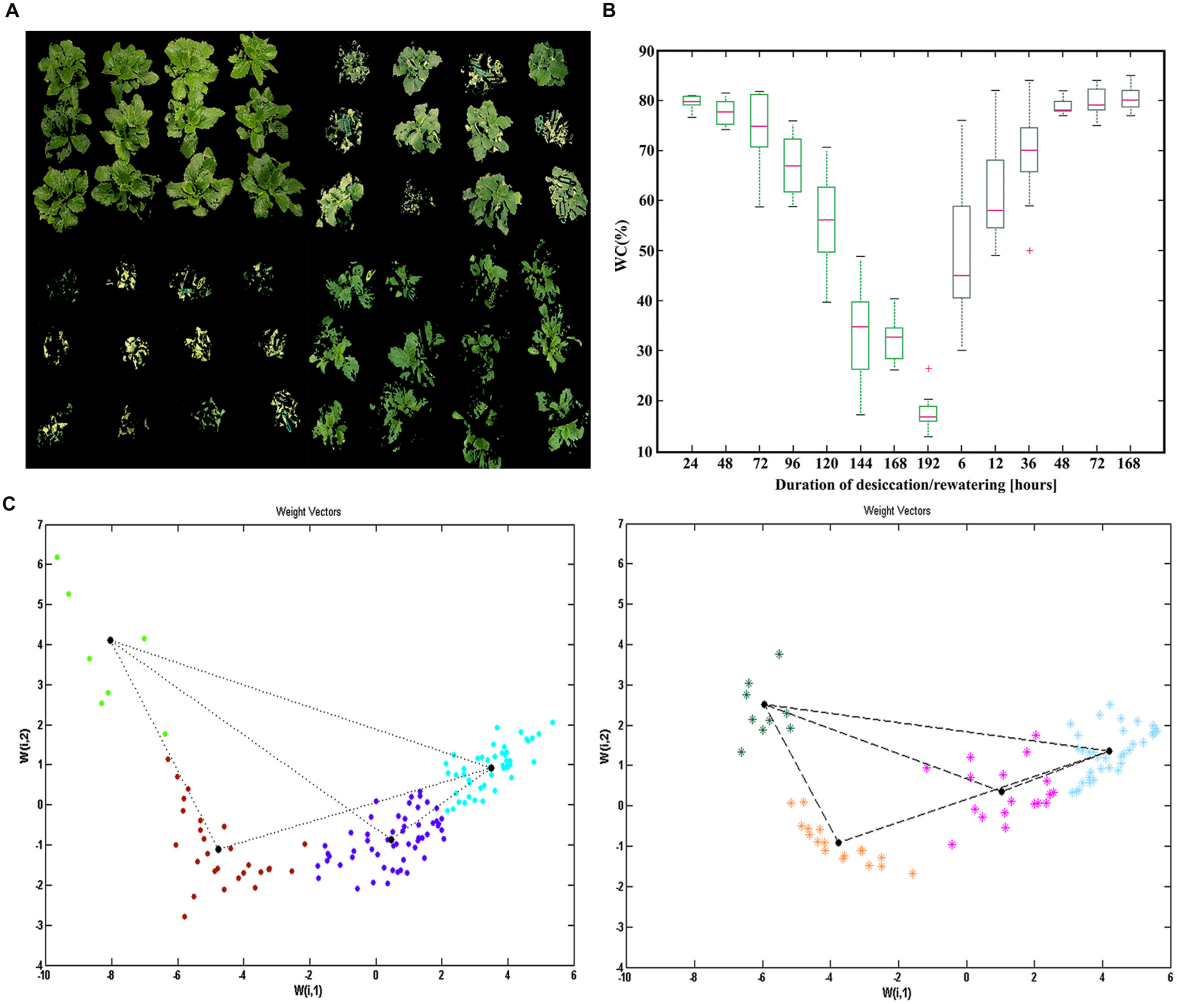
**Evaluation and quantification of plant water status during desiccation and recovery of *Haberlea rhodopen*sis. (A)** Drying and recovery of different plants (upper left, Control plants; upper right 120 h of desiccation; lower left, 192 h of desiccation; lower right 36 h of recovery). **(B)** Box plots of changes in water content during desiccation (blue) and recovery (green). **(C)** Self organizing map (SOM) visualization of the classified samples according to the four pre-defined neurons for desiccation and recovery. The positions of the scores (leaf samples) derived after Principal Component Analysis (PCA) transformation are clustered in different colors with the neurons according to their weights defined with the two weight vectors, for desiccation (left) and for recovery (right), respectively.

In the present study, the leaf water status was evaluated by comparing leaf WC and prompt chlorophyll fluorescence. WC started to decline sharply after 72 h desiccation. On the average, it took about 8 days for the *H. rhodopensis* plants to reach full desiccation – 16% WC, starting from about 80% WC. After 10 days at fully dried state, irrigation was performed. WC recovered rapidly during the first 6 h upon irrigation, and almost regained the initial values after 48 h (**Figure 1B**), but the plants needed seven days to fully recover after rewatering (**Figure 1A**).

At every sampling point we observed a significant heterogeneity in leaf WC (**Figure 1B**). Therefore, we decided to apply a non-destructive sampling, using JIP-TEST derived from both PEA devices, to better define the sampling points. According to PCA, the first two principal components explained approximately 75 and 15% of the variance of JIP parameters of the leaf samples (Supplementary Table S1). Most of the parameters with largest contribution to the separation of the leaf samples in PC 1 are mainly related to quantum yields (ph Po, phiEo and phiRo) and performance indexes (PI_ABS,_ PI_total_). The residual orthogonal part of the variation in PC 2 was related mainly to the fluxes per reaction centers (ABS/RC, REo/RC, ETo/RC, TRo/RC). We used regression analysis to show the relation of the JIP variables with different contribution in PCA and WC (Supplementary Table S1). After evaluation of variance in the PC, we created a one layer SOM with four pre-specified neurons (**Figure 1C**). As a result, the leaf samples were centered according to the neurons, to four pre-specified clusters in the same pattern as in the PCA space. We defined the average values of leaf WC and JIP TEST parameters of each plant state referred to different water stress levels, using the data points with the most proximal weight positions (9–12 measurements) to each neuron (Supplementary Table S2). As a result, we were able to pinpoint several intermediate states, characterized both by fluorescent transients and corresponding WC (Supplementary Table S2). We defined a control (C)-79% WC; two intermediate states of desiccation D1-59% WC and D2-38% WC and one state for fully dried plants D3-16% WC; an intermediate recovery (R1)-73% WC point and full recovery (R2)-81 % WC point. In addition, the measurements with handy PEA enabled us to define reliable markers [phi Po^light^ (ΦPSII) and phi Eo^light^] in light-adapted plants (Supplementary Figure S1).

All further sampling for various analyses was performed non-destructively, based on the JIP TEST outputs and changes in the PSII functionality. In this way, we were able to measure fluorescence transients and to determine the physiology state of about 50 leaves from various intact plants in a period of 60 min. According to their classification in the SOM (**Figure 1C**), they were related to the corresponding states of desiccation and recovery.

### Photosynthetic Performance and Organization of Photosynthetic Apparatus of *H. rhodopensis* during Selected States of Desiccation and Recovery

**FIGURE 2:**
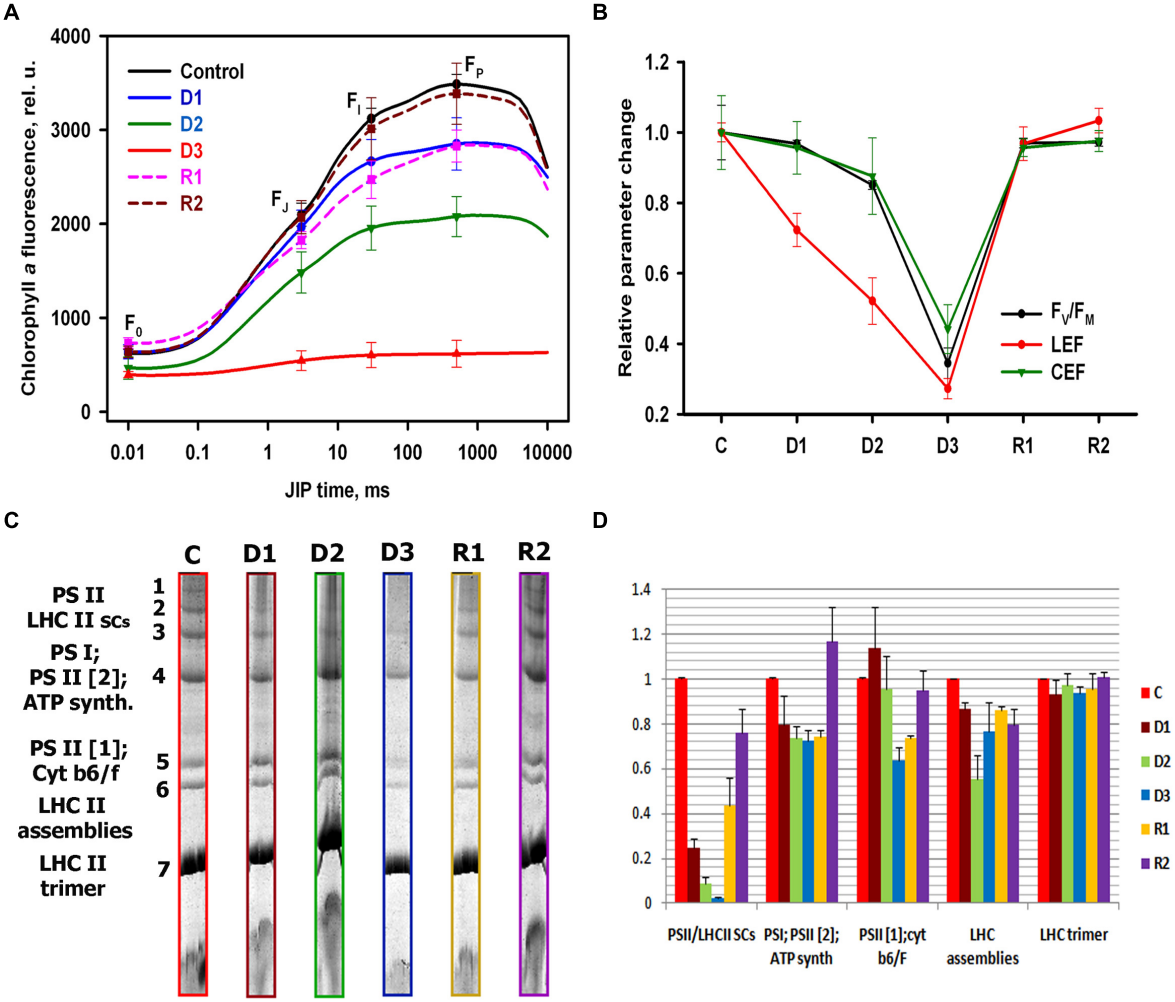
**Photosynthetic performance and organization of photosynthetic apparatus of *H. rhodopensis* during selected states of desiccation and recovery. (A)** Representative averaged OJIP curves and water content (WC), respectively, from different samples classified with SOM, corresponding to different stress states of plants. State C: watered plants (80%), state D1: moderate desiccation (60%), state D2: severe desiccation (38%), state D3: dry plants (16%), state R1: intermediate recovery (73%), state R2: full recovery (81%). The SD was calculated from 9 to 12 measurements, respectively. **(B)** Quantification of Fv/Fm, linear and Cyclic electron flow at selected states of desiccation and recovery. The signal abundance is normalized to control state. The SD was calculated from 6 measurements, respectively. **(C)** BN-PAGE separation of thylakoid protein complexes at selected states of desiccation and recovery after solubilization with *n*-dodecyl-β-D-maltoside (DDM). **(D)** Densitometry quantification of the resolved photosynthetic complexes normalized to control levels. The SD was calculated from three independent gels.

The OJIP parts of the PF transients depicted all parameters exhibit pronounced decrease reflected by the decreasing of P-level (*F*_P_), during drought stress, followed by the reversible increasing during recovery (**Figure 2A**). The calculated quantum yields and performance indexes (Supplementary Table S2), derived from the averaged OJIP curves (**Figure 2A**) show that the maximum quantum yield for primary photochemistry (phi Po), do not change significantly during C–D1 state transition, followed by a moderate decrease during D1–D2 transition, and a more drastic change during D2–D3 transition. On the other hand, we observed a pronounced decrease of the quantum yield for electron transport beyond QA (phi Eo) and quantum yield for reduction of end electron acceptors at the PSI acceptor side (φRo), which clearly coincided with the decrease of leaf WC (Supplementary Table S2). The absorption by PSII antenna pigments (ABS/RC), trapping energy (TRo/RC, ETo/RC) and the electron transport per fully active PS II reaction center increased during drought (Supplementary Table S2), indicating an inactivation of the PSII reaction centers. Indeed, we showed that the efficiency of PSII during water stress corroborates previous studies on the inhibition of CO2 fixation (Georgieva et al., 2007) in parallel with the WC. It appears that the well-established relationship between the PSII activity and CO_2_ assimilation (Genty et al., 1989) is still present during water deprivation in *H. rhodopensis.* We evaluated LEF from fluorescence-derived analysis, of plants in light-adapted state (**Figure 2B**) as commonly done in several publications for resurrection plants (Georgieva et al., 2007, 2010; Ingle et al., 2008; Beckett et al., 2012). CEF was estimated by changes in the redox state of P_700_ upon illumination with far red light of previously dark-adapted material (**Figure 2B**). By assessing the initial rate of P_700_ oxidation in far red during the different steps of water deprivation and rehydration (Supplementary Figure S2), we were able to quantify the CEF: LEF ratio and to compare it with changes in linear flow (from the ΦPSII) and in the PSII efficiency, here evinced from the Fv/Fm parameter We found that during the selected states of stress, the maximum quantum yield for primary photochemistry (Fv/Fm), and LEF decreased in an identical way as the measured quantum yields derived from OJIP curves. A smaller decrease was found in the CEF efficiency, suggesting that this process was more resistant to the applied water stress (**Figure 2B**). Our results from BN-PAGE showed bands representing the typical organization of the photosynthetic apparatus in plants: PSII dimer (PSII [2]); PSII monomer (PSII [1]); cytochrome b_6_f complex (Cytb_6_f); light-harvesting complex II (LHCII) assemblies; LHCII, as well as high molecular weight supercomplexes consisting of different forms of PSII (LHCIIsc), (**Figure 2C**). During the transition from C–D1 state, the higher molecular weight complexes were disassembled and the bands corresponding to PSII-LHCIIsc were reduced (**Figure 2D**). This decrease of PSII-LHCIIsc continued up to the D3 state. At full dry state all supercomplexes were disassembled and the abundance of PSII dimers also declined. In addition decline of cytochrome b_6_f complex content in thylakoids has been observed during D2–D3 state transition. During rewatering, the supercomplexes were partially recovered at R1 state and fully recovered at R2 state. These observations might suggest a relation between changes in the relative abundance/functional assembly of PSII and the main electron transport carrier-cytochrome b_6_f complex (**Figure 2D**) and the relative contribution of PSII-driven LEF and PSI -driven electron flow during desiccation (**Figure 2B**).

### NMR Metabolic Profiles during Desiccation and Recovery

**FIGURE 3:**
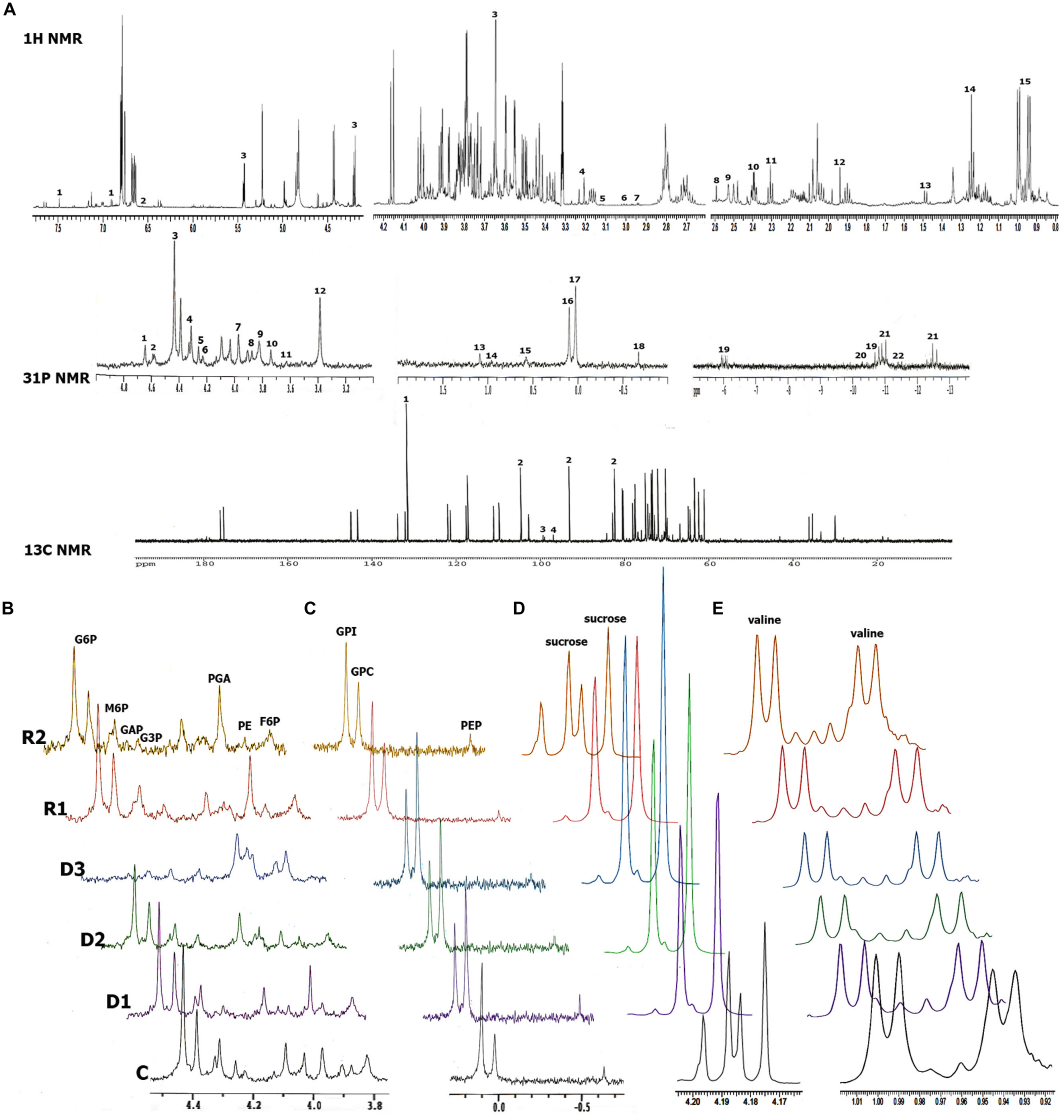
**NMR metabolic profiles of *H. rhodopensis* during selected states of desiccation and recovery. (A)** Representative NMR spectra with the identified compounds. ^1^H NMR spectra: 1-caffeic acid; 2-fumarate; 3-sucrose; 4-choline; 5-ethanolamine; 6-GABA; 7-aspartate; 8-succinate; 9-citrate; 10-glutamate; 11-acetic acid; 12-alanine; 13-ethanol; 14-valine. ^31^P NMR spectra: 1-dihydroxyacetone phosphate (DHAP);2-phosphogluconate (P-gluconate); 3-α glucose 6-phosphate (G6P); βG6P; 4-α mannose 6-phosphate (M6P); βM6P; 5-glyceraldehyde phosphate (GAP); 6-glycerol 3-phosphate (G3P); 7-phosphoglycerate (PGA); 8-phosphatydilethanolamine; 9-Fru 6 P; 10-AMP; 11-NADPH; 12-Phosphocholine (P-choline); 13-glycerophosphoglycerol (GPG); 14 -GPGPG; 15-glycerophosphoethanolamine (GPE); 16-glycerophosphoinositol (GPI); 17-glycerophosphocholine (GPC); 18-phosphoenolpyruvate (PEP); 19-adenosine triphosphate (ATP); 20-adenosine diphosphate (ADP); 21-UDP-glucose; 22-NADP. C13 NMR spectra: 1-maleate; 2-sucrose; 3-fructose; 4-glucose. **(B,C)** Alignment of regions with glycolytic and lipid compounds, respectively, from ^31^P NMR during stress. **(D,E)** Alignment of sucrose and valine regions, respectively, from ^1^H NMR spectra during stress. States of dehydration and recovery are as defined in **Figure 1**

**FIGURE 4:**
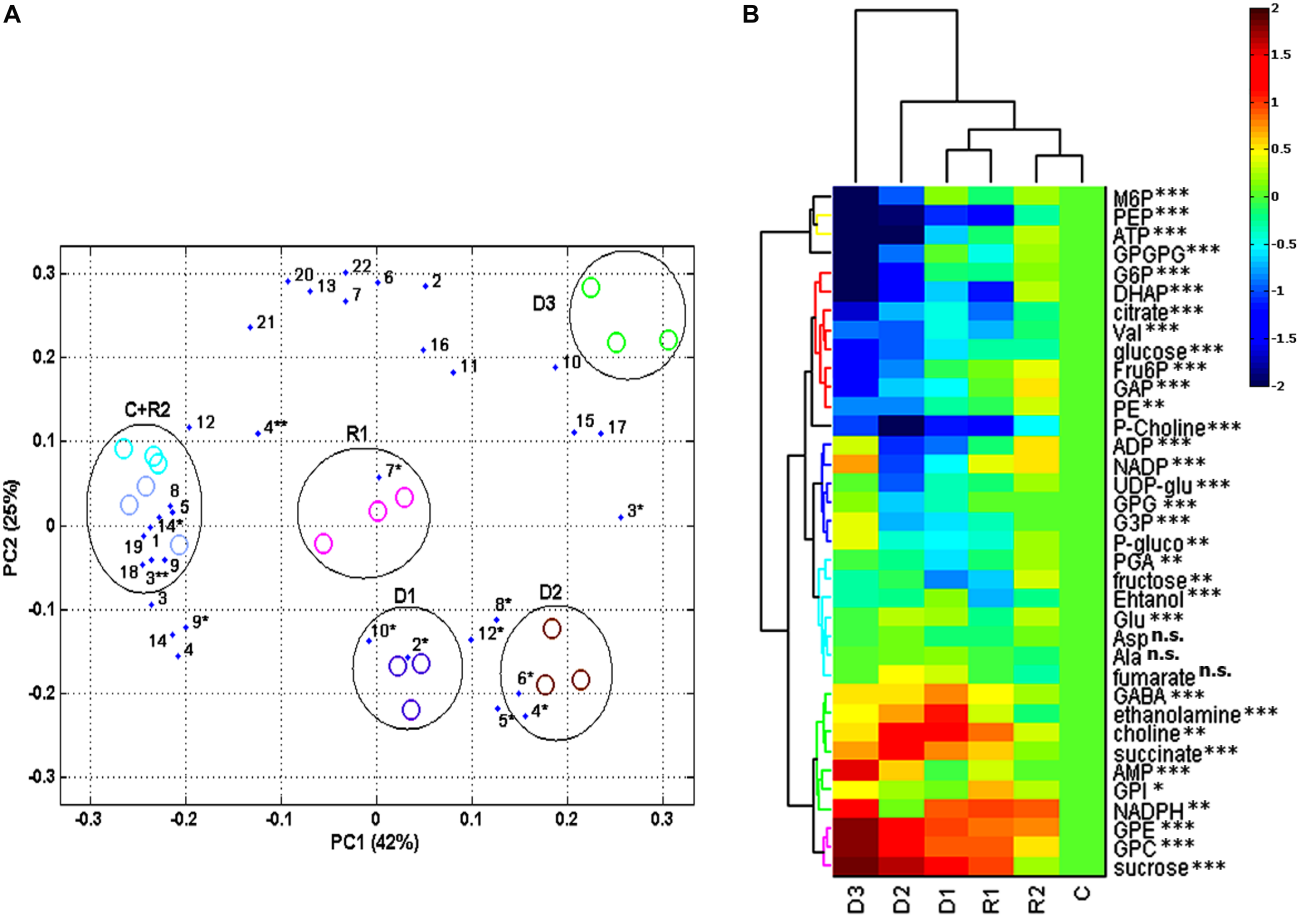
**Statistical analysis and visualization of changes in metabolomics data of *H. rhodopensis* during selected states of desiccation and recovery.** States of dehydration and recovery are as defined in **Figure 1 (A)** PCA biplot of the data, contains the measured metabolites identified in *H. rhodopensis* as loadings represented as numbers, evaluated in samples at various states (as defined in **Figure 1**) as scores represented with ovals: electric blue for (C) and light blue for R2; violet for D1 and pink for R1; brown for D2 and green for D3. The compounds numbers correspond to assignment of metabolites in NMR spectra (see **Figure 3**). ^∗^correspond to metabolites identified with ^1^H NMR; ^∗∗^correspond to metabolites identified with ^13^C NMR; compounds identified with ^31^P NMR lack asterisks. **(B)** Hierarchical cluster analysis (HCA)/Heat map clustering and visualization of dynamics of the quantified compounds represented by their log2 transformed averaged meanings, normalized to control levels. *P*-values for significance of changes during stress treatments were assigned to each metabolite as: n.s.- non significant, ^∗^- significant, ^∗∗^-very significant, ^∗∗∗^- extremely significant.

Combining ^13^C, ^31^P, and ^1^H NMR, we were able to identify and quantify a total of 36 metabolites during dehydration and recovery in *H. rhodopensis* (**Figure 3**, Supplementary Table S3). Almost all of the compounds represented in *H. rhodopensis* showed significant changes during stress (Supplementary Table S3). Sucrose, valine, fructose, glucose, citrate, ethanol, and gucose-6-P were with the highest amounts in the extracts (Supplementary Table S3). The performed PCA explained 70% of the total variance of metabolites within the samples by the two main components. The first group of metabolites (loadings) with highest contribution in PC1 is: phosphoenolpyruvate (PEP), glucose, dihydroxyacetone phosphate (DHAP), glucose 6-phosphate (G6P) and ATP (**Figure 4A**, loading numbers: 18, 4^∗∗^, 1, 3, 19) as positive ones and the second group of main negatives are: glycerophosphocholine (GPC), sucrose and glycerophosphoethanolamine (GPE), (**Figure 4A**, loading numbers: 17, 3^∗^, 15). Therefore, the leaf samples (scores), in C and R2 state, grouped in the negative scale, are separated from the others due to their highest content of glycolytic intermediates and ATP and low levels of sucrose, GPC and GPE (**Figure 4A**, scores) on the contrast to the fully dry leaf samples which are extremely in positive scale (**Figure 4A**, scores). The PC2 separate mainly D3 state in the positive scale and D1 and D2 in the negative from the other samples (**Figure 4A**, scores), according to the higher content of NADP, ADP, glycerophosphoglycerol (GPG) and glycerol 3-phosphate (G3P) in D3 (**Figure 4A**, loading numbers: 22, 20, 13, 6), and the higher content of ethanolamine and gamma-aminobutyric acid (GABA) in D1 and D2, respectively (**Figure 4A**, loading number: 5^∗^, 6^∗^). The HCA/Heat map (**Figure 4B**) showed the same group pattern of the treatments and metabolite changes as PCA, but provided more detailed visualization of the metabolite changes during stress. The metabolites of the first main cluster – bis(glycerophospho)glycerol (GPGP), mannose 6-phosphate (M6P), ATP, and PEP showed a drastic decrease from D1–D3 stages (**Figure 4B**, rows, top cluster). G6P, DHAP, citrate, valine, glucose, fructose 6-phosphate (F6P), glyceraldehyde phosphate (GAP), phosphatidylethanolamine (PE), decreased immediately after the onset of water deficit and are grouped in the second main cluster (**Figure 4B**, rows, red group). Phosphocholine (P-choline) is separated from this cluster according to the lower levels in R2. The third main cluster is presented by two different groups. The first one (blue) – ADP, NADP, UDP-glucose (UDP-glu), GPG, G3P, and 6-phosphogluconate groups metabolites with more or less decreased concentrations in D1 and D2 states, with a tendency of accumulation during D2–D3 state transition. The second group (light blue) combines metabolites with relatively stable concentrations during drought stress – 3-phosphoglycerate (PGA), fructose, ethanol, glutamate, aspartate, alanine, and fumarate. The metabolites with increased concentration during drought stress represent the fourth main cluster (**Figure 4B** rows, bottom cluster). GPC, GPE, sucrose (contributing for the separation of D3 in PC1 positive scale) are grouped in pink. The second group (green) of this cluster combines metabolites with heterogeneity in their accumulation during drought. NADPH, glycerophosphoinositol (GPI) and AMP increased from D2–D3. Succinate, choline, ethanolamine, and GABA accumulated during D1 and D2 states and decreased in D3 state, thus explaining the separation of these states in PC2. After rewatering (R1), the metabolites recovered their abundance to levels close to D1 state. At R2 state, the contents of the metabolites were more or less close to control (C) state.

**FIGURE 5:**
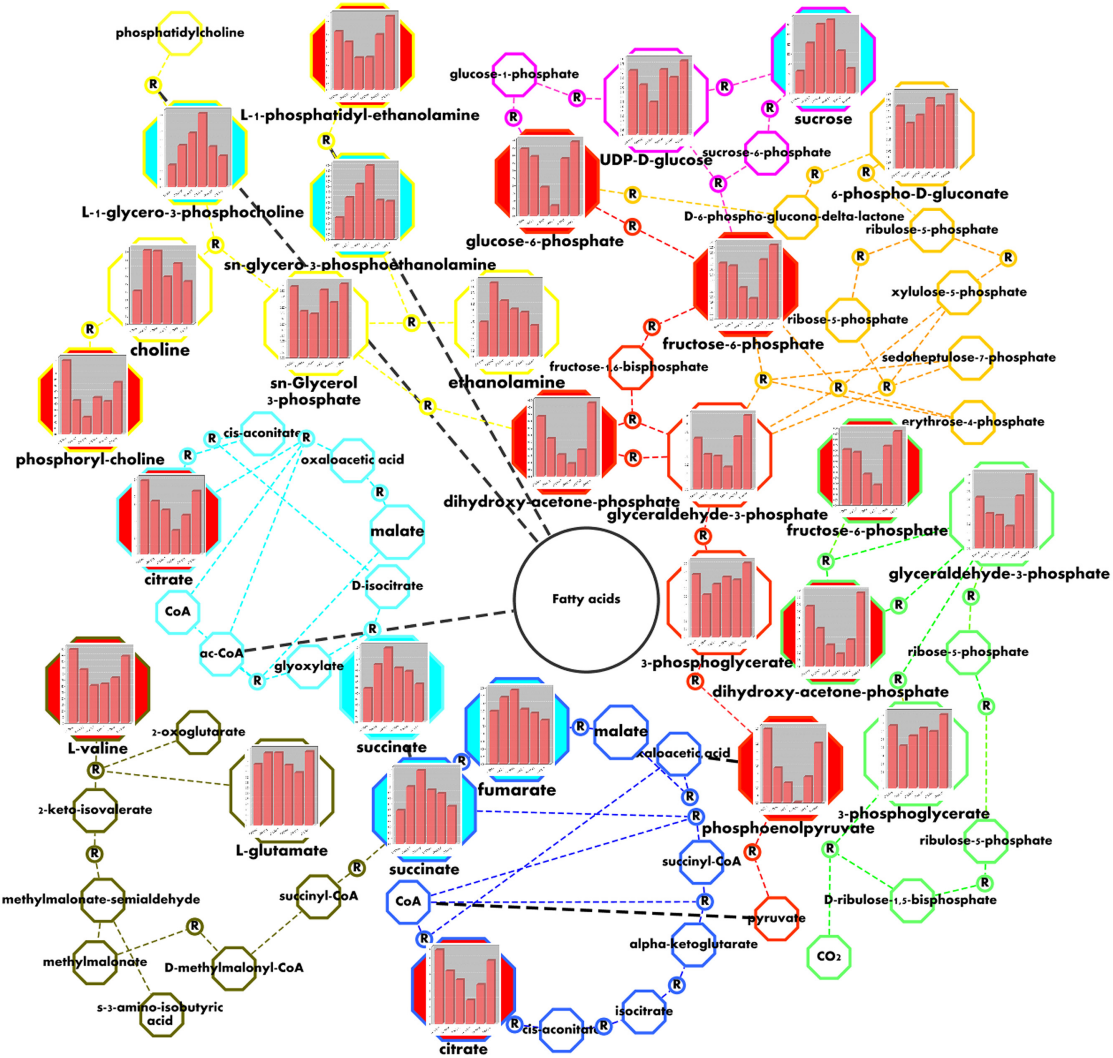
**Visualization of metabolic changes in of *H. rhodopensis* during selected states of desiccation and recovery**. Values correspond to the averaged meanings of metabolites in the context of sucrose biosynthesis (pink), glycolysis (red), Calvin cycle (light green), pentose phosphate pathway (orange), glycerophospholipid metabolism (yellow), valine and glutamate degradation (dark green), glyoxylate cycle (light blue), and TCA cycle (dark blue) metabolic pathways. Bars from left to right represent the C, D1, D2, D3, R1, and R2 states, respectively, as defined in **Figure 1** The colored nodes represent the metabolites changes during drought with Pearson correlation above 0.8. The blue ones represent the metabolites increasing and the red ones represent those with decreasing concentrations. Circled R represents enzymatic reaction.

The compounds quantified here fall in several plant metabolic pathways (**Figure 5**). The accumulation of sucrose until D3 state was accompanied with declining of the levels of UDP-glu in D1 and D2 and an increase in D3 state (**Figure 5**, pink). The dynamics of F6P and G6P also showed an intensive consumption from D1–D3 stages (**Figure 5**, red), suggesting the change of direction of glycolysis toward sucrose biosynthesis during these transitions. The slightly increased level of PGA in D3 could be due to the organelle compartmentation of Calvin cycle (**Figure 5**, green). The increased levels of phosphogluconate (**Figure 5**, orange), suggest up-regulation of pentose phosphate pathway, which could serve as a supplying unit of erythrose-4-phosphate. Altogether with PEP, they could be used for accumulation of polyphenols through shikimic acid pathway. The observed degradation of PE in *H. rhodopensis* during drought was accompanied with the increased levels of GPE during the course of drought stress (**Figure 5**, yellow). We observed same trends for GPC (**Figure 5**, yellow) suggesting that phosphatidylcholine (PC) could also be subjected to degradation. The levels of choline and ethanolamine increased in D1 and D2 state in relation with the observed degradation of PE and the accumulation of GPE and GPC (**Figure 5**, yellow). The pronounced degradation of valine (**Figure 5**, dark green), accompanied by the increase of succinic acid (**Figure 5**, dark green) and fumaric acid (**Figure 5**, dark blue) could be related to the supply of carbon sources for gluconeogenesis *via* succinate and the TCA cycle. The decrease of citric acid (**Figure 5**, dark blue) could be due to the exchange of tricarbons between the citric acid and cytosol for gluconeogenesis.

## Discussion

A very important prerequisite to evaluate the dynamics of plant response to drought is the establishment of a reliable and reproducible evaluation system. Experiments with potted plants often have problems with the heterogeneity of samples (Verslues et al., 2006; Oliver et al., 2011) due to the irregular drying of substrate in the vessels (Verslues et al., 2006).

The most widely used method for estimation of the water status of leaves is the evaluation of the WC. Quantification of this parameter by traditional methods is a slow process but more importantly, this method is destructive. Here, we show that a non-destructive, fast, and reproducible method can be used to evaluate the leaf water status of *H. rhodopensis* and to define discrete intermediate states during drought stress and recovery. This method is based on the prompt fluorescence changes in intact plants and competitive machine learning (Maldonado et al., 2003; Goltsev et al., 2012). The results, derived from the OJIP test (**Figure 2A**) corresponding to different stress states are in good agreement with the previously published studies obtained with detached leaves of *H. rhodopensis* (Strasser et al., 2010). The energy flux trapped to the reaction center then converted to redox energy for reducing the electron transport chain which lead ultimately to CO_2_ fixation, decrease in almost identical way with the decreasing of WC. The decrease of LEF between the PSs and CO_2_ assimilation has been reported previously in H. *rhodopensis*, (Georgieva et al., 2007). Here, combining prompt fluorescence and adsorption spectroscopy, we found that in contrast to this decrease, the CEF around PSI shows high activity until the full dry state (**Figure 2B**). Several events have been suggested, which could affect the electron transfer activity and the electron flow mode. The changes in osmolarity induced by water restriction could modify the diffusion of the soluble electron carriers, namely ferredoxin and plastocyanin (Cruz et al., 2001). Also the specific degradation of the PSII supercomplex could lead to a situation where this complex can no longer contribute electrons to the other complexes of the photosynthetic electron flow chain. Previous studies compared the PSII supercomplex reorganization in shade and sun populations of *H. rhodopensis* during drought (Sárvária et al., 2014). In agreement with these observation, we show here (**Figures 2C,D**) that degradation during the selected states of desiccation (confirmed by the increased ratio of absorbing chlorophylls per active RC during drought) could lead to a situation where this complex can no longer contribute electrons to the other complexes of the photosynthetic electron flow chain. The specific decrease of cytochrome b_6_f; PSII band (**Figures 2C,D**) showed that the regulation of inhibition of electron transport could be contributed also by the cytochrome b6f complex/PSII, although to a lesser extent than the PSII supercomplex, as reported also in *Craterostigma pumilum* (Charuvi et al., 2015). Therefore, CEF could be simply enhanced because reduced PSII activity could no longer compete with electron injection by PSI for the reduction of the cytochrome complex electron donors. This maintenance of CEF in contrast to the pronounced decreasing of the LEF in *H. rhodopensis* during drought stress could have a photoprotective role as already shown in other resurrection plant species (Huang et al., 2012), by maintaining an active PSI (which is not susceptible to photoinhibition). Moreover, by maintaining some photosynthetic ATP synthesis without net consumption, CEF could avoid a complete deprivation of ATP, thereby contributing, together with respiration, to fuel specific processes required for plant survival during the late states of desiccation.

Parallel with the observed changes in photosynthetic machinery, all identified metabolites showed significant changes during desiccation and recovery, leading to global changes in primary metabolism. In our study, at desiccation of *H. rhodopensis*, ATP decreased reaching undetectable levels at fully dry state (Supplementary Table S3). Possibly, ATP was intensively used for energy dependent metabolic reactions (such as sucrose synthesis) during drought stress. We speculate that the detected levels of ATP in D2 state, where the LEF is inhibited, in parallel with the intensive sucrose accumulation, could be due to the unchanged activity of CEF in this state. In addition, we did not observe drastic decrease of NADP/NADPH ratio during stress, probably due to the photoprotective functions of the LEF/CEF switch. However, the relative increase of NADPH in D3 stage could be a result of processes related to over-reduction of electron transport chains in some extend, during D2–D3 state transition. Next, we show that the tricarbons from glycolysis and Clavin cycle decrease in parallel with the light reaction of photosynthesis. Interestingly, only the level of PGA showed slight increasing during drought. We suggest that its relatively high levels of PGA in chloroplasts could function as storage pool ensuring fast recovery of photosynthesis during rewatering.

It is widely accepted that accumulation of sucrose at desiccation is among the main protective mechanisms for *H. rhodopensis* (Muller et al., 1997; Djilianov et al., 2011; Gechev et al., 2013; Moyankova et al., 2014). In good agreement with the previous data, our results show that sucrose is massively accumulated during the late stages of desiccation, where the changes in the structure of PSII are already complete (**Figure 2C**). In this respect, we confirm the recently proposed link between the accumulation of sucrose and changes in the non-bilayer phase separation in thylakoid membranes, PSII arrays and HII phases during dehydration of homoiochlorophyllous resurrection plants (Charuvi et al., 2015). To date, the accumulation of sucrose is often explained by fast degradation of starch (Gechev et al., 2013; Moyankova et al., 2014). However, the role of starch as a sole source for sucrose accumulation has been already questioned earlier (Muller et al., 1997) since it is difficult to find a clear correlation between the fast consumption of starch at the early stages and the accumulation of sucrose in the very late stages of desiccation (Muller et al., 1997; Moyankova et al., 2014; Suguiyama et al., 2014). It has been proposed that considerable part of sucrose accumulation at late stages of desiccation in *Ramonda nathaliae* (a close relative of *H. rhodopensis*) was due to gluconeogenesis (Muller et al., 1997). Our present data strongly support this hypothesis, since we observed significant consumption of glycolytic intermediates in parallel with the accumulation of sucrose (**Figure 5**).

In contrast of the relatively low abundance of most identified amino acids, the value of valine before stress was almost similar to that of sucrose (Supplementary Table S4). During desiccation, valine declined twice and recovered under rewatering. The stress-related degradation of this particular amino acid was confirmed by the accumulation of 3-aminoisobutyric acid (secondary product of valine and leucine degradation) and accompanied by the degradation of leucine as observed in our previous study (Moyankova et al., 2014). It appears that like non-resurrection plants (Diebold et al., 2002; Schuster and Binder, 2005) *H. rhodopensis* needs branched amino acids as alternative carbon sources for sucrose biosynthesis during stress.

Together with PC, PE is the main phospholipid in the endoplasmic reticulum, Golgi apparatus, and cell membranes (Douce et al., 1987). During stress, the quantity of PE declined twice (**Figure 5**). In this respect, we observed pronounced accumulations of GPE and GPC due to the degradation of PE and probably PC. Such processes of cell membrane lipid degradation are reported in *Ramonda serbica* (Quartacci et al., 2002). It could be due to their oxidative damage and/or the activation of phospholipase-1 activity, as reported in other resurrection plant species *Craterostigma plantagineum* (Frank et al., 2000). Pronounced accumulation of lysolipids namely acyl-GPC and acyl-GPI, was related with membrane lipid degradation in *Sporobolus stafianus* (Oliver et al., 2011). We suggest that after the phospholypase activity resulting in accumulation of lysolipids, the lysophospholipases activity reduces the toxic properties of acyl-GPC for the membranes (Atkinson et al., 2008; Howland and Parikh, 2010) and releases fatty acids and glycerophosphodiesters. Such lipid degradation could ensure fast and reversible accumulation of glycerophosphodiesters (Stålberg et al., 2008), which could be involved in osmoprotection of cells in *H. rhodopensis* as previously suggested in yeasts (Gallazzini et al., 2008). On the other hand the free fatty acids could also be used as alternative carbon sources through the glyoxalate cycle, which is in good agreement with their previously reported degradation at desiccation (Moyankova et al., 2014). It is known that the main quantity of phosphatydylglycerol is located in chloroplast membranes and diphosphatidylglycerol is a mitochondrial lipid marker (Douce et al., 1987). In our study, the levels of GPG and GPGPG (derivatives of phosphatydylglycerol and cardiolipin) remained stable during water stress (Supplementary Table S3). This suggests that the degradation of phospholipids of these two organelles is highly restricted in *H. rhodopensis* while a degradation of MGDG in chloroplasts has been shown in *Craterostigma plantagineum* (Gasulla et al., 2013).

The accumulation of succinate and fumarate at early stages of desiccation (D1–D2; **Figure 5**), as already shown (Gechev et al., 2013), in parallel with the decrease of valine and the free fatty acids (as products of lipid turnover), confirm the maintenance of respiration activity and could be due to the entering of these alternative carbon sources in the TCA cycle. Moreover, recent transcriptomics analyses performed on *H. rhodopensis* showed overexpression of succinate dehydrogenase (Georgieva et al., 2012) as the main enzyme involved in the respiration and processes of carbon recycling from fatty acids and valine via succinate (**Figure 5**). On the other hand, the observed decreasing of citric acid during drought highlights the utilization of the tricarbons derived from the respiration.

Widely discussed osmolytes (e.g., proline or glycinebetaine; Ashraf and Foolad, 2007; Chen and Murata, 2011) were not found during our NMR studies which is in agreement with t the previously reported absence or very insignificant amounts of these compounds in *H. rhodopensis* (Gechev et al., 2013; Moyankova et al., 2014). It is known that GPC is involved in osmoprotection of cells (Gallazzini et al., 2008). Obviously, along with the accumulation of sucrose, the accumulation of GPC (Supplementary Table S3) could ensure additional osmoprotection for the survival of *H. rhodopensis* during extreme drought.

## Conclusion

The present study provides a new approach for evaluation of plant water status by non-destructive sampling based on the dynamics of photosynthetic performance under desiccation stress. This method establishes a connection between the photosynthetic performance with all other evaluated processes and enabled us to reproducibly determine the physiological state of numerous leaf samples from various intact plants of *H. rhodopensis* in a short period of time. Furthermore, we showed that CEF remains active almost till full dry stage in contrast to the LEF, due to the changes in protein organization of photosynthetic machinery. We suggest that, this activity could have a photoprotective role and together with respiration prevent a complete drop of ATP, utilized for specific energy-dependent processes during desiccation. Here, we confirmed the hypothesis for downregulation of photosynthesis in the anhydrobiotes, as well as the need of respiration activity for accumulation of osmoprotectants. We suggest that alternative carbon sources for the massive sucrose accumulation at late stages of drying are provided by high initial amounts of valine and lipid degradation (through the fatty acid catabolism). Such lipid degradation could ensure fast and reversible accumulation of glycerophosphodiesters, which could be involved in osmoprotection of cells in *H. rhodopensis*.

## Author Contributions

The experiments were conceived and designed by: PM, GF, RB, NR, DD. The experiments were performed by: PM, GF, RB, SS, AMB, KA, DM, VK, DZ, SB. The data were analyzed by: PM, GF, RB, SS, VG. The paper was written by: PM, GF, MT, MF, NR, DD.

## Conflict of Interest Statement

The authors declare that the research was conducted in the absence of any commercial or financial relationships that could be construed as a potential conflict of interest.
