# Supplementary material for: In vivo spectroscopy and NMR metabolite fingerprinting approaches to connect the dynamics of photosynthetic and metabolic phenotypes in resurrection plant Haberlea rhodopensis during desiccation and recovery
Source: Front Plant Sci. 2015 Jul 21;6:564. doi: 10.3389/fpls.2015.00564 (PMC4508511; doi:10.3389/fpls.2015.00564)
Supplement: Supplementary file 1 [file Table_1.PDF]

Table S1. PC 1 and 2 loading values of the fluorescence parameters derived from JIP test and their correlation with water content

Fluorescence parameters	Description	Desiccation						Recovery					
		M-PEA			Handy PEA			M-PEA			Handy PEA		
		PC 1 (75%)	PC2 (14%)	Corr. with WC	PC 1 (72%)	PC2 (13%)	Corr. with WC	PC 1 (75%)	PC 2 (11%)	Corr. with WC	PC 1 (98%)	PC 2 (0.99 %)	Corr. with WC
ϕPo = 1-F0/FM	maximum quantum yield of primary photochemistry (at t = 0)	0.26	0	0.96	0.26	-0.14	0.86	0.26	0	0.75	0	0	0.94
ϕEo = (1-F0/FM)(1-VJ)	quantum yield of electron transport (at t = 0)	0.25	0.08	0.92	0.26	0.01	0.91	0.26	0.11	0.96	0	0	0.93
ϕRo = (1-F0/FM)(1-VI)	quantum yield for reduction of end electron acceptors at the PSI acceptor side (RE)	0.24	0.15	0.89	0.23	0.01	0.87	0.26	0.08	0.96	0	0	0.88
ABS/RC = (1-γRC)/γRC	absorption flux (of antenna Chls) per RC	-0.17	0.45	-0.44	-0.16	0.43	-0.63	-0.19	0.34	-0.68	0	0	-0.89
TRo/RC = M0(1/VJ)	trapping flux (leading to QA reduction) per RC	-0.15	0.47	0.97	-0.07	0.43	0.77	-0.18	0.34	0.63	0	0	0.66
ETo/RC = M0(1/VJ)(1-VJ)	electron transport flux (further than QA–) per RC	-0.14	0.48	0.98	-0.02	0.54	0.45	-0.13	0.52	0.87	0	0	0.58
REo/RC = M0(1/VJ)(1-VI)	electron flux reducing end electron acceptors at the PSI acceptor side, per RC	-0.17	0.28	0.51	-0.16	0.41	0.58	-0.19	0.39	0.86	0	0	0.35
RC/CSo = ϕPo . (VJ/M0) . F0	density of RCs (QA-reducing PSII reaction centers) approximated by F0	0.26	0.03	-0.59	0.26	-0.07	-0.75	0.17	0.16	-0.47	0.05	-0.11	-0.41
ABS / CS0 ≈ F0	absorption flux (of antenna Chls) per excited cross-section approximated by F0	0.2	-0.25	0.8	0.23	-0.04	0.89	0.08	-0.35	-0.41	0.05	-0.39	0.87
TR0 / CS0 = ϕPo . F0	trapping flux (leading to QA reduction) per excited cross-section approximated by F0	0.25	-0.04	0.9	0.26	-0.05	0.94	0.25	-0.07	0.43	0.1	-0.32	0.89
ET0 / CSo = ϕPo . (1-VJ) . F0	electron transport flux (further than QA–) per excited cross-section approximated by F0	0.26	0.05	0.92	0.26	0.07	0.92	0.26	0.06	0.95	0.06	0	0.94
REo/CS0 = ϕPo . (1-VI) . F0	electron flux reducing end electron acceptors at the PSI acceptor side per excited cross-section approximated by F0	0.25	0.1	0.9	0.25	0.06	0.86	0.25	0.02	0.94	0.02	-0.05	0.9

RC/CSM = ϕPo . (VJ/M0) . FM	density of RCs (QA-reducing PSII reaction centers) per excited cross-section approximated by FM	0.26	0.12	0.9	0.26	0.06	0.79	0.26	0.08	0.9	0.3	0.23	0.79
ABS/CSM ≈ FM	absorption flux (of antenna Chls) per excited cross-section approximated by FM	0.25	-0.01	0.94	0.26	0.06	0.98	0.26	-0.02	0.95	0.64	-0.4	0.98
TRo/CSM = ϕPo . FM	trapping flux (leading to QA reduction) per excited cross-section approximated by FM	0.26	0.06	0.99	0.26	0.07	1	0.26	0.08	0.97	0.59	-0.01	1
ETo/CSM = ϕPo .(1-VJ) . FM	electron transport flux (further than QA–) per excited cross-section approximated by FM	0.25	0.13	0.94	0.26	0.15	0.95	0.26	0.15	0.98	0.35	0.72	0.97
REo/CSM = ϕPo . (1-VI) . FM	electron flux reducing end electron acceptors at the PSI acceptor side per excited cross-section approximated by FM	0.25	0.16	0.93	0.25	0.16	0.94	0.26	0.14	0.97	0.09	0.03	0.97
PIABS = γRC /(1-γRC.ϕPo/(1-ϕPo) . ψEo/(1-ψEo)	performance index (potential) for energy conservation from exciton to the reduction of intersystem electron acceptors	0.21	0.22	0.83	0.24	0.19	0.94	0.24	0.23	0.93	0.01	0.02	0.89
PItotal = PIABS . δRo/(1- δRo)	performance index (potential) for energy conservation from exciton to the reduction of PSI end acceptors	0.21	0.23	0.9	0.22	0.2	0.95	0.23	0.23	0.97	0	0.01	0.97

Additional parameters defined some of parameters from the Table.

$M_0 \equiv [(\Delta F/\Delta t)_0]/(F_M - F_{50\mu s})$ – Approximated initial slope (in ms^{-1}) of the fluorescence transient normalized on the maximal variable fluorescence F_V;

$\psi_{Eo} \equiv ET_0/TR_0 = (1-V_J)$ – Efficiency/probability that an electron moves further than Q_A

$\delta_{Ro} \equiv RE_0/ET_0 = (1-V_I)/(1-V_J)$ – Efficiency/probability with which an electron from the intersystem electron carriers is transferred to reduce end electron acceptors at the PSI acceptor side (RE);

$\gamma_{RC} = Chl_{RC}/Chl_{total} = RC/(ABS + RC)$ – Probability that a PSII Chl molecule functions as RC

Table S2. Averaged absolute meanings with standard error of the JIP parameters and WC respectively, correspond to different stress states. 6-9 measurements for each state are averaged based on their distance from each neuron in the calculated SOM.

Parameter	C	D1	D2	D3	R1	R2
**W.C.**	79.11±0.94	58.85±1.94	38.37±0.72	15.62±0.62	73.38±2.05	81.32±0.65
**phi(Po)**	0.8±0	0.77±0.01	0.42±0.02	0.03±0	0.68±0.03	0.81±0
**phi(Eo)**	0.48±0.01	0.37±0.02	0.2±0.01	0.01±0	0.32±0.03	0.48±0.01
**phi(Ro)**	0.1±0	0.06±0	0.05±0	0±0	0.12±0.01	0.13±0
**ABS/RC**	1.97±0.03	2.17±0.05	3.73±0.23	55.8±8.46	3.11±0.18	2.33±0.04
**TRo/RC**	1.58±0.02	1.67±0.03	1.54±0.04	2.2±0.13	2.14±0.16	1.88±0.03
**ETo/RC**	0.94±0.03	0.81±0.04	0.74±0.03	1.3±0.05	0.99±0.09	1.12±0.02
**REo/RC**	0.2±0.01	0.13±0.01	0.23±0.01	0.36±0.02	0.36±0.03	0.31±0.01
**RC/CSo**	335.26±5.98	312.93±6.02	133.25±9.2	7.92±1.22	259.83±11.87	293.85±3.55
**ABS/CSo**	659.45±12.8	678.65±19.62	478.5±18.5	347.2±10.08	746.18±21.93	685±14.39
**TRo/CSo**	530.21±8.54	522.96±15.8	205.06±15.89	11.6±0.38	506.2±25.23	553.18±10.8
**ETo/CSo**	314.98±9.64	252.72±12.29	96.29±4.04	4.64±0.62	237.52±23.27	328.32±6.52
**REo/CSo**	67.52±2.22	40.92±1.83	29.43±1.35	1.54±0.46	89.48±5.82	92.28±2.78
**RC/CSm**	1721.17±40.48	1380.42±56.11	241.24±24.31	8.2±1.27	901.96±98.91	1530.87±22.82
**ABS/CSm**	3377.82±42.07	2987.05±121.58	849.75±54.29	359.2±10.31	2531.64±216.9	3564.25±59.23
**TRo/CSm**	2718.36±40.35	2308.39±109.27	371.25±39.58	12±0.4	1785.45±214.65	2879.25±47.64
**ETo/CSm**	1609.82±29.4	1109.82±58.09	172±12.02	4.8±0.64	844.91±134.6	1710.5±37.12
**REo/CSm**	345.27±7.89	180.56±10.6	52.5±3.82	1.6±0.47	302±29.68	480.75±15.02
**PI(abs)**	30.76±0.92	15.17±1.17	1.99±0.21	0.01±0	8.43±1.83	26.78±1.33
**PI(tot)**	8.41±0.31	2.95±0.24	0.91±0.12	0.01±0	4.65±0.54	10.46±0.56

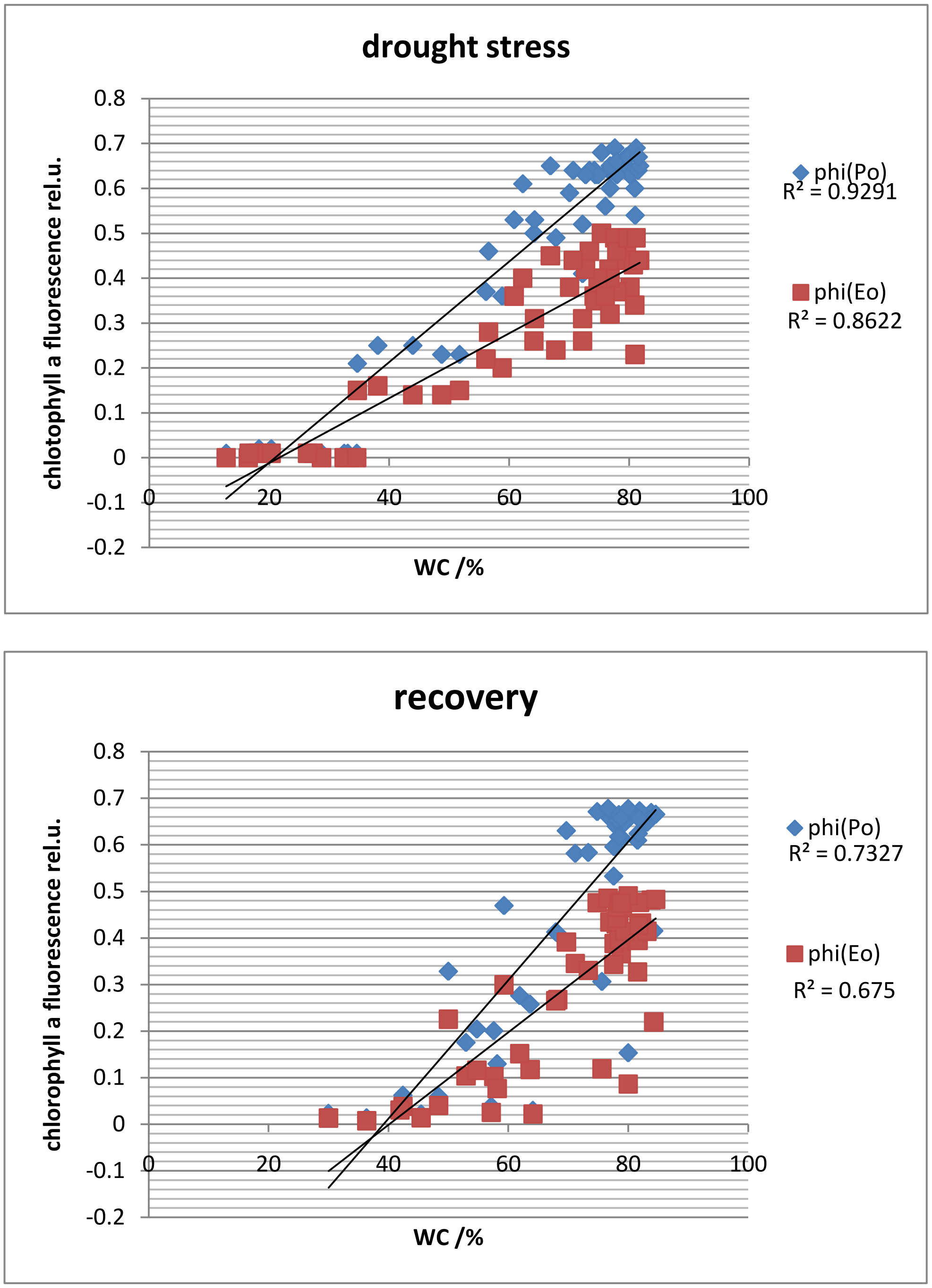

Figure S1. Dynamics of phi Po^{light} (ΦPSII) and phi Eo^{light} in Light-adapted plants

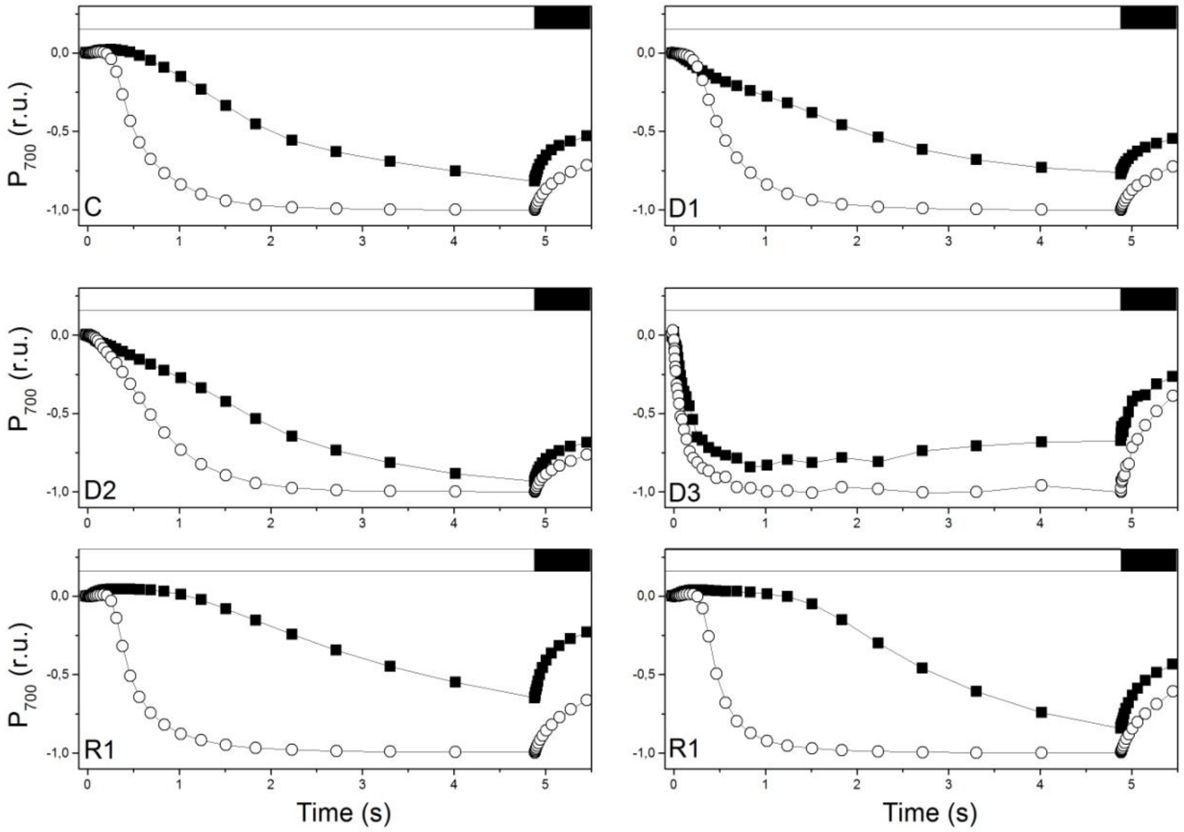

Figure S2 Oxidation of P700 during illumination of dark-adapted and light acclimated eaves of *H. rhodopen*sis leaves with far-red light.
Data show representative traces measured in leaves form plants at different desiccation stages (see text for description). Solid squares: dark adapted leaves. Open circles: light acclimated leaves (150 µ mol photons m-2 s-1) for 10 minutes and dark adapted for 1 min before P700 oxidation kinetics were measured with far-red illumination. Open box: far red light on. Solid box: far red light off.
Clearly, P700 oxidation is biphasic in dark-adapted leaves. This biphasicity was interpreted by the presence of two PSI populations: a fast one (the fast phase representing PSI centers involved in linear electron transfer (*Joliot and Joliot 2005*). There, P700 oxidized by the linear pathway is not re-reduced by electron transfer from PSII, which is poorly excited by far red light. The second population (the slow phase), would represents PSIs involved in cyclic flow. For these complexes, net P700 oxidation is slowed, because of the recycling of electrons through PSI turnover in far red light.

Table S3. Metabolic profiles of different stages of desiccation. Metabolites were quantified using internal standards described in materials and methods for each NMR method. P values from one-way Anova are calculated for each metabolite. Values were obtained from a three biological and three technical replicates and are given as mean ± SD.
* - ^{1}H NMR; **- ^{31}P NMR; ***-^{13}C NMR

compound	chemical shift/ppm	p value	Concentration µmol/gDW± st.error					
			**C**	**D1**	**D2**	**D3**	**R1**	**R2**
fumarate*	6.55	0.0779	1.74±0.14	2.18±0.24	2.42±0.09	1.8±0.11	1.67±0.05	1.44±0.19
sucrose*	4.18;5.41	6.23E-13	60.47±2.21	137.79±2.08	190.26±2.33	229.19±15.08	116.58±1.68	69.24±1.53
choline*	3.21	0.0022	0.41±0.02	0.92±0.16	0.91±0.08	0.59±0.06	0.75±0.1	0.53±0.02
ethanolamine*	3.12	9.96E-04	1.47±0.13	3.15±0.03	2.38±0.17	2.02±0.21	1.88±0.18	1.33±0.03
GABA*	3.01	2.00E-05	1.43±0.03	2.46±0.2	2.05±0.1	2.07±0.14	1.93±0.01	1.64±0.07
Asp*	2.96	0.3857	1.8±0.16	1.58±0.07	1.88±0.07	1.73±0.21	1.62±0.06	1.9±0.05
succinate*	2.6	6.44E-04	0.58±0.05	1±0.11	1.28±0.15	0.94±0.01	0.88±0.15	0.66±0.04
citrate*	2.52	1.26E-04	8.88±0.33	6.36±0.63	5.3±0.34	2.83±0.54	4.71±0.2	7.6±0.82
Glu*	2.46	7.90E-04	1.13±0.03	1.34±0.02	1.35±0.03	1.12±0.11	0.98±0.03	1.37±0.03
Ala*	1.48	0.1601	1.3±0.1	1.45±0.11	1.38±0.04	1.32±0.01	1.3±0.01	1.18±0.05
Ehtanol*	1.25	1.72E-05	5.13±0.05	5.81±0.13	4.45±0.3	4.18±0.32	3.09±0.04	4.41±0.19
Val*	0.94;0.99	8.52E-06	59.71±3.73	43.48±0.92	30.57±0.95	31.79±2.56	36.61±1.41	54.27±3.56
DHAP**	4.64	2.67E-06	0.43±0.05	0.27±0.02	0.16±0.02	0.09±0	0.19±0.04	0.53±0.04
P-gluco**	4.59	0.0011	0.27±0.02	0.19±0.01	0.2±0.02	0.36±0.02	0.2±0.03	0.32±0.02
αG6P;βG6P**	4.39;4.37	5.27E-09	4.4±0.11	3.92±0.22	1.88±0.2	0.66±0.01	3.77±0.5	4.87±0.35
αM6P;βM6P**	4.31;4.32	9.61E-14	1.34±0.06	1.5±0.19	0.69±0.1	0.01±0	1.2±0.09	1.53±0.11
GAP**	4.26	1.78E-04	0.31±0.04	0.21±0.03	0.2±0.03	0.13±0.02	0.32±0.03	0.45±0.04
G3P**	4.23	9.89E-04	0.32±0.01	0.21±0.02	0.2±0.02	0.42±0.02	0.25±0.04	0.33±0.04
PGA**	4.13	0.0053	0.76±0.06	0.51±0.04	0.64±0.03	0.73±0.06	0.69±0.06	0.9±0.06
PE**	4.03	0.0017	0.94±0.08	0.78±0.03	0.52±0.04	0.52±0.06	0.89±0.22	1.19±0.09
Fru 6 P**	3.82	1.18E-06	0.5±0.05	0.48±0.06	0.28±0.02	0.18±0.01	0.54±0.04	0.67±0.03
AMP**	3.73	5.36E-04	0.29±0.04	0.29±0.02	0.45±0.06	0.83±0.11	0.38±0.05	0.3±0.04
NADPH**	3.46	0.0014	0.14±0.01	0.27±0.03	0.16±0.02	0.36±0.03	0.28±0.05	0.27±0.04
P-Choline**	3.68	8.39E-06	1.21±0.05	0.54±0.08	0.28±0.02	0.6±0.04	0.53±0.05	0.85±0.12
GPG**	1.072	5.31E-04	0.57±0.07	0.44±0.03	0.36±0.02	0.64±0.03	0.58±0.03	0.58±0.02
GPGPG**	0.96	9.40E-10	0.26±0.02	0.27±0.04	0.14±0.02	0.01±0	0.19±0.03	0.31±0.03
GPE**	0.579	6.31E-04	0.21±0.04	0.4±0.03	0.52±0.07	0.69±0.06	0.37±0.05	0.36±0.03
GPI**	0.091	0.0194	2.36±0.29	2.52±0.23	2.7±0.18	3.33±0.18	3.72±0.28	2.83±0.25
GPC**	0.014	4.88E-07	1.36±0.09	2.59±0.14	3.36±0.39	4.62±0.17	2.52±0.07	1.93±0.11
PEP**	0.658	5.79E-13	0.99±0.05	0.47±0.04	0.26±0.03	0.01±0	0.35±0.04	0.8±0.07
ATP**	-5.94	3.17E-12	0.65±0.05	0.42±0.05	0.12±0.01	0.01±0	0.6±0.07	0.79±0.07
ADP**	-10.49	1.67E-04	0.26±0.02	0.13±0.02	0.12±0.02	0.33±0.05	0.23±0.03	0.37±0.02
UDP-glu**	-10.9	3.34E-04	0.67±0.06	0.52±0.02	0.34±0.02	0.68±0.08	0.61±0.05	0.78±0.09
NADP**	-11.3	7.42E-05	0.2±0.03	0.14±0.01	0.1±0.02	0.33±0.04	0.27±0.03	0.29±0.03
glucose***	61.444	6.61E-07	11.48±0.84	6.02±0.67	3.88±0.71	26.72±2.53	56.49±2.28	57.3±2.8
fructose***	68.4853	0.0029	20.76±1.75	11.48±1.77	19.1±1.9	17.28±1.4	13.1±2.3	26.61±1.95
